# Pathology and Treatment of Chronic Cervical Endometritis

**Published:** 1888-10

**Authors:** J. W. McLaughlin

**Affiliations:** Austin


DANIEL'S

### Texas Medical Journal.

A Representative Organ of the Medical Profession, and an Exponent of Rational Medicine; devoted to the Organization, Advancement and Elevation of the Profession in Texas.

Published Monthly.—Subscription $2.00 a Year.

Vol. IV. AUSTIN, OCTOBER, 1888. No. 4.

### Original Articles.

CONTRIBUTED EXCLUSIVELY TO THIS JOURNAL.

*The Articles in this Department are accepted and published with the understanding that we are not responsible for, nor do we indorse the views and opinions of the writers by so doing.*

### PATHOLOGY AND TREATMENT OF CHRONIC CERVICAL ENDOMETRITIS.

By J. W. McLaughlin, M. D., Austin.

[Read at Austin District Medical Society and ordered printed in Daniel's Texas Medical Journal.]

IN compliance with your wishes, which were made known to me by our Secretary, the following remarks upon the "Pathology and Treatment of Chronic Cervical Endometritis," are submitted for your consideration and for discussion.

The frequent occurrence of this disease, its variableness, conflicting opinions regarding its pathology, and the difficulties attending its successful treatment render this subject one of great practical interest to the physician, particularly as such cases constitute a very large part of his gynecological practice.

As pathology is based upon anatomy, and treatment upon pathology, I will ask your indulgence, while I preface what I have to say by a brief review of some of the leading facts relating to the anatomy of the cervix uteri.

The cervix is that portion of the uterus, which is situated below the internal os; it is above one inch in length, a little less in its transverse diameter, and about one half as great in its antero-posterior diameter.

It is spindle shaped, lies entirely below the peritoneum, and is described as consisting of two parts, a supra-vaginal and an infra-vaginal portion; the former lies between the internal os and the fornix or vault of the vagina; the latter is that portion situated below the vaginal vault, and is usually termed the portio-vaginalis.

The supra-vaginal portion lies within the parametrical space and is surrounded by cellular tissue and a network of blood-vessels, nerves, lymphatics and ganglia. The infra-vaginal portion is invested by a reflection of the vaginal mucous membrane.

The principal structures which enter into the formation of the cervix uteri, are, connective tissue, muscular tissue, mucous membrane, blood-vessels, lymphatics and nerves; the connective tissue largely predominates, and is found in greater relative proportion than in the body of the uterus.

The cervical mucosa is thicker and firmer, and differs from that of the uterus in its arrangements and glandular structure; it is corrugated, thrown into folds, and in the virgin presents an arborrescent appearance, from which it derives its name, "arbor vitæ." It is covered by a single layer of cylindrical epithelium which is ciliated upon the surface of the plica.

The cervical mucous membrane is richly supplied with racemose glands, branching ducts with dilated ends, lined with cubical epithelium and surrounded by a plexus of blood-vessels. These glands secrete a clear mucus of an alkaline reaction, their ducts open on the ridges or plica, and also in the depressions of the mucosa. The cylindrical epithelium which covers the cervical mucosa, abruptly terminates at the external os, forming a boundary line, beyond which we find the vasculæ papillæ and pavement epithelium of the vagina.

The uterine artery chiefly furnishes the cervix with its blood supply; it arises from the anterior division of the pudic, and passes along the base of the broad ligaments to a point opposite the external os, when it sharply bends upwards by the side of the cervix, to finally unite by many anastomosing branches with the descending branches of the ovarian artery.

During its course it gives off a great many lateral branches to the vagina, cervix and body of the uterus. It is claimed that the lateral branches of the uterine and ovarian arteries are distributed to the various segments of the uterus in such a manner that it is impossible for congestion of the uterine vessels to result, as formerly claimed, from flexions of the womb.

The veins and lymphatics from the cervix, are large and numerous, and surround this segment of the womb with a network of interlacing vessels. The uterus receives its nerves chiefly from the sympathetic system.

Chronic cervical endometritis is one of the most frequent diseases of women, and unfortunately, is one of the most difficult to cure.

It is essentially a chronic inflammation of the cervical mucosa, and its muciperous glands, associated frequently with certain definite pathological changes in the connective tissue and muscularis of the cervix. These morbid changes may extend to the general endometrium, and other tissues of the womb, or may begin in these, and secondarily involve the cervix.

Different appearances and conditions of the cervix will often be revealed by a specular examination or by digital touch in different cases of chronic cervical catarrh, or in the same case at different stages of the disease; these differences result, in part, from many etiological facts, but mainly depend upon which of the tissue elements of the cervix predominate in the morbid action; for example, inflammation of the cervical mucosa with active proliferation of its cylindrical epithelium will present appearances differing in many respects from that condition in which the muciperous glands with proliferation of their glandular epithelium dominate. Again both of these present different features from that condition caused by hyperplasia of the connective and muscular tissue; which has been so perfectly described by Thomas, and called by him "areolar hyperplasia."

Rarely however, do we find these morbid changes confined singly to one of the tissues of the cervix, that is, superficial epithelium, glandular structure or connective tissue; generally one of these specific affections predominates, whilst the other two complicate, or one is the cause or sequel of the other. Of these conditions, isolated cervical catarrh is most frequently observed. We then find the inflammation limited to the mucosa and its epithelial covering; the mucous membrane is reddened, swollen, and its foldings are greatly increased; the swollen membrane can frequently be seen protruding from the patulous os; it is exceedingly vascular, and often bleeds from the slightest touch. The exaggerated foldings of the mucosa with its papillæ enlarged and reddened, can be seen through the epithelial covering and has given to this condition the name of papillary degeneration, or granular degeneration, the former when the foldings, and the latter when the papillæ are most conspicuous. This condition is best seen and beautifully marked, where isolated cervical catarrh is associated with laceration of the cervix, and ectropion of its lips.

But the inflammation will rarely stop here. Very soon it involves the muciperous glands and their cubical epithelium. There will then result an increased secretion from these glands and closure of their ducts; the first greatly adds to the existing leucorrhea, the second, which arises from pressure on the ducts exerted by the hypertrophied mucosa gives rise to a retention of the secretion, and produces that pathological condition called ovula nabothii or nabothian follicles; when these attain a large size, they are called retention cysts.

These cysts sometimes form polypi, or their contents become purulent, and abscesses result. The smaller cysts or nabothian follicles are however most frequently observed; from rupture of these follicles, and other causes, the cubical epithelium which lines them, and which is possessed of the power of rapid proliferation, will, starting from these ruptured follicles, rapidly spread itself over the mucosa, or displacing the squamous, epithelium of the portio vaginalis, pass beyond the external os, and form red patches with well defined abrupt limits upon the vaginal portion of the cervix. The difference in color of these patches, contrasted with the healthy, and much paler color of the vaginal mucous membrane, gave rise to the opinion that these patches were ulcerations, and they were so called until a recent period. They in fact, consist of an extension of the glandular tissue of the cervical mucosa by proliferation over the portio vaginalis, constituting, in fact, a new growth of glandular tissue.

Associated with this condition there is frequently found upon the surface of the portio vaginalis a number of clear vesicular bodies, firm to the touch, and which can be felt by the examining finger like shot under the surface. This condition is known as follicular degeneration, whilst the small, hard bodies are the nabothian eggs which have already been referred to. These bodies are formed throughout the diseased mucosa or upon the portio vaginalis in consequence of the extension of the glandular tissue to the vaginal mucosa. When the cervix is extensively invaded by them and especially when they are of usual size, we have what is known, as cystic degeneration. Associated with these morbid changes, or existing as a part of a general chronic metritis, or endometritis, or perhaps, as a result of traumatisms, there will be found a condition of the cervix in which its connective tissue is greatly increased, whilst the cervix itself is congested and succulent, or firm or hard, with increase in its length and its antero posterior diameter; this condition is called by Thomas "areolar hyperplasia." It is proper to state that the pathology of chronic cervical catarrh, and its results are, in many respects, still open questions. The description of pathological changes which have been submitted, are I believe, those which are most generally accepted by pathologists as correct.

The treatment of chronic endometritis, like its pathology, is not yet placed beyond dispute. It would needlessly occupy your time, and perhaps weary you, to present even an abstract of the various remedies and plans of treatment advised at different times, together with all the minute directions as to methods and manner of use. The usual method is to discuss treatment under the two heads of constitutional and local. We will depart from this time-honored custom in the present instance, and confine our attention to the local treatment. I do not wish to convey the idea that constitutional treatment is not necessary; on the contrary, it is all-important; but to discuss it in all its features would lengthen this paper beyond your patience, besides, the general therapeutical indications are so well understood by the profession that the time at our disposal can be better employed in discussing the local measures of treatment. Local treatment will have for its aim the normal restoration of pathological tissue by alterative or caustic applications, or the arrest of pathological changes; or in case neither of these results can be accomplished, the destruction or removal of pathological tissues is demanded.

Chief among the many alterative and caustic remedies which are applied to the diseased cervical mucosa, are the following: Tincture iodine, carbolic acid, or the two remedies combined in iodinized phenol, pyroligneous acid, chromic acid in solution of 25 to 50 per cent., chloride zinc in solution of 25 per cent., nitrate silver, full strength, or modified nitric acid and acid nitrate mercury. Hot water vaginal irrigations, or applications to portio vaginalis of tr. iodine, boro glyceride or glycerine, or interstitial injections into cervix, of tr. iodine or f. e. ergot; besides vaginal suppositories and a host of vaginal injections.

I do not think it would be interesting or profitable to enter into a description of the method, manner or frequency of applying these remedies; but there are certain general rules relating to this subject, and to that of the surgical treatment of chronic cervical catarrh, of such great importance that I deem it proper to ask your attention, and I briefly enumerate those which occur to me:

1. No caustic or irritating application should be made to the cervix during the presence of pelvic inflammations, cellulitic or peritonitic.

2. Successful application cannot be made through a tight os uteri, or contracted cervical canal; these conditions should be first overcome by dilatation, divulsion, or the knife.

3. The field of operation should be cleared of mucus or morbid secretions before applications are made.

4. Retention cysts or ovula nabothis should be opened previous to the other treatment.

5. The stronger caustics, and especially nitric acid, should not be applied often, or at short intervals, because of their tendency to cause contraction or atresia of the cervical canal.

6. Chromic acid, mercurics and arsenic, when used in full strength, are liable to cause dangerous toxic symptoms.

7. All cervical lacerations should be repaired by Emmet's operation, after the disease has been cured.

8. When we consider the intimate anatomical relations which exist between the cervix uteri and the broad ligaments, with their numerous blood-vessels and lymphatics, it should impress us with the important fact that no surgical operation upon the cervix should be performed, and no caustic application which would leave a raw, granulating or absorbing surface should be made, except under strict antiseptic rules.

Many cases of chronic cervical endometritis cannot be cured by caustic or alterative applications; the inflamed and hypertrophied mucosa, the glandular proliferation and hyperplastic increase of connective tissue will continue to exist, or perhaps increase, notwithstanding the most active local application of caustics and their therapeutical adjuncts. Under these circumstances, a cure can be accomplished only by a total destruction of the diseased mucosa and its glands. The means at our command which can be safely used to do this, are the sharp curette, the knife, and the continuous galvanic current.

Thomas uses the sharp curette, as follows: He introduces it into the cervical canal as high up as the internal os, then by a downward scraping forcibly removes the diseased mucous membrane from every part of the canal. Fritsch supplements the removal of the mucosa by the curette with a subsequent application of strong nitric acid to every portion of the scraped surface.

Schrœder's operation consists in removing, with the knife, the diseased mucosa follicle, and substituting for the mucous membrane of the cervix that of the vagina. It is done as follows:

Split the cervix bilaterally up to the vaginal fornix. At the upper limit of the incision, make a transverse incision through the mucosa of both the anterior and posterior lips which have thus been formed. Introduce a sharb pointed knife under the mucosa at the tip of one of the cervical lips, and push it—the knife—up to the transverse incision. Then cut right and left, thus entirely removing the diseased membrane. Do the same with the other lip, and then turn the lips inwards, bending them upon themselves so as to cover the denuded surface. The lips should be fastened in this position by properly introduced sutures.

The latest, most novel, and, it would seem from published reports of cases, a very successful method of treating chronic endometritis and uterine hyperplasia, whether general or cervical, is by electricity. The favorable results obtained by Rockwell and others, by the Farradic current, has for some time been the property of the profession; the more recent labors of Engleman and Apostoli, with the continuous galvanic current, are not so well known. The excellent results which they claim to have secured from the current, stamps it as a most reliable and powerful therapeutical agent.

Among the many properties claimed for it are, that it improves nutrition; it stimulates; it allays pain; it causes absorption, and it controls hemorrhage. In the treatment of those morbid changes which occur in the female sexual organs, the application of galvanism requires but little time; it is safe, practically painless, easy of application, and often curative.

It must be remembered that the negative and positive poles are very dissimilar in their action, do different kinds of work, and are often antipodal in their therapeutical properties.

The negative pole is more active than the positive, the chemical effect being greater; it is the painful, irritating caustic pole, and its tendency is to destroy—to produce hemorrhage.

The positive pole is anæsthetic, the least painful, causes absorption, and its tendency is to check hemorrhage.

Now there are two recognized methods of using the constant galvanic current in the treatmeut of chronic endomitritis and uterine hyperplasia, One method, that of galvanic puncture, is championed by Engleman; the other method is that preached by Apostoli. Inasmuch as endometritis and hyperplasia of the uterus are pathogenetically the same when confined to cervix, it is reasonable to assume that the curative results claimed for electricity in the former would also be obtained from its use in the latter troubles. Engleman says: "In chronic metritis and hyperplasia we utilize the absorbent and electrolytic properties of the negative galvanic current,and the chemical action of the galvanic pole; also the contracting and stimulating cffect of the Faradic currents of quality and low tension. These cases are frequently accompanied by a scanty menstrual flow and dysmenorrhœa, hence the hemorrhagic tendencies of the negative pole are of service, as well as its electrolytic and cauterizing properties. The most effective treatment, if there is no contra indication, is negative electropuncture; passing the platinum needle into the indurated tissue parallel to the uterine canal, connecting this with the negative pole of the battery, placing the positive dispersing pole upon the abdomen, and using a current of from 50 to 150 milliamperes. The larger stylet may also be inserted, or four or five needles at a time surrounding the os, all connected with one and the same negative pole. If amenorrhœa, painful menstruation or narrowing of the canal, especially in case of endometritis, accompany the hyperplasia, it is first of all important to remedy these conditions, and cauterization takes the place of puncture; that is, the uterine sound connected with the negative pole is used in the cavity, whilst the positive pole is in connection with the dispersing plate of the abdomen."

"If weaker currents, from 40 to 60 milliamperes, are used, electropuncture of the uterus may be repeated every third or fourth day; the application or currents of from 120 to 150 milliamperes should be from four days to one week apart, as they are accompanied by slight destruction of tissue, which at first leaves an open canal, but at the end of that time nothing but a slight depression in the cervical tissue, at the point of puncture remains."

Grandin, in a concise description of Apostoli's method, as described by him in his recent monograph, says: "Apostoli's new method aims at utilizing in the greatest degree the chemical and trophic action of electricity, in order to destroy the deseased endometrium and to exert a derivatory effect upon the uterus. The routine method generally resorted to for the cure of chronic endrometritis and of hyperplasia, one and all, aim at altering the vicious nutrition, at causing absorption of the hypoplastic tissue and at eliminating the pathological process in the endometrium. These methods, it is the experience of all, are slow in action, often ineffective, and where efficient this is only after the lapse of considerable time. For this reason Apostoli has rejected them, and after prolonged experience advocates the substitution of electricity as being, where proper precautions are taken, uniformly safer, and. where the current is properly utilized, as being followed by excellent results. For the purpose of application of this new method Apostoli uses special electrodes, which we will briefly describe : The external electrode, he claims, must possess such qualities as will enable very intense currents to be used, without causing discomfort or injury |to the woman; and these qualities are obtainable by means which diminish to the greatest possible degree the resistance of the skin.

In galvanization of the female genital organs it is ever to be borne in mind, that they are not especially sensitive to the action of the current, no matter how intense, and that the problem simply is to neutralize the action of the current at its external electrode, which, as we have elsewhere stated, is accomplished by increasing the surface over which the current is disseminated externally.

Soft, moist, adhesive potters clay is the material, which in Apostoli's hands, has answered best for the external electrode, and by means of it he has been enabled to triple and quadruple the maximum current which it was formerly deemed safe to employ. This clay must be plastic and soft, in order to allow of accurate adaption to the surface; it must retain its humidity, and the layer must be uniform in thickness, and not too thick; else it offers additional resistance to the passage of the current.

Apostoli prepares this electrode as follows : In a wooden or metal mould about 1½ centimeter high, he places a layer of wet tarlton, and then fills the mould with the clay exactly to the top. This clay mass should be large enough to cover the entire abdomen, and before being applied, the skin should be examined for abrasions, and if one is found it should be covered with collodion, else the skin will be cauterized at this point. Connection is made with the battery by means of a metal plate fused to the rheophone, and this plate is pressed gently into the clay.

Apostoli's internal electrode is, in shape and size, like the ordinary uterine sound; the handle is about four inches long and is constructed of celluloid, which is not only a poor conductor of electricity, but also aseptic, in that it does not absorb. This handle slips over the electrode proper, so that the surface not in use is thorougly insulated. The sound electrode is constructed of platinum, an agent which is not corroded when the positive pole is used. Before inserting the electrode into the uterus, it should be carefully disinfected, and should be introduced like the ordinary uterine sound.

In utilizing the chemical galvano-caustic action it is essential to remember the different effects of the positive and negative poles. In cases of hyperplasia or of endometritis, where hemorrhage and leucorrhœa are marked symptoms, the internal electrode is to be connected with the positive pole, and in the reverse instances, the negative. Such being the special electrodes which Apostoli utilizes, and such being the reasons for the choice for one or the other pole, as the internal one, it remains to describe the method of application of the current, the intensity to be selected, the duration of the seance and the frequency of repetition.

The patient should occupy the dorsal position, and be cautioned to keep absolutely quiet; the electrode is inserted to the fundus, guided by the finger in the vagina, and the celluloid sheath is pushed down to the cervix so as to thoroughly protect the vagina.

This electrode is held and steadied by the hand during the entire seance, the layer of clay is uniformly adapted to the abdomen, and the metal plate is pressed gently into it. The rheophones are then connected, and after waiting a while, till all reaction induced by the insertion of the sound has disappeared, the current is very gently and gradually turned on, avoiding all shock.

At the first seance Apostoli has found it advisable not to exceed 100 milliamperes; but later, when he has tested the tolerance of the patient, he aims as high as 200, even 250 milliamperes.

In general, the seance should last from three to ten minutes, the duration depending on the nature of the individual case, and on the sensibility of the patient. The true guide to the dosage is the rule never to cause the patient much pain. The seance is ended, even as it began, gently, and avoiding much shock.

After the seance, rest should be enjoined for several hours, and the patient will very likely suffer from uterine colic for a certain period, and for this reason the post operative period is much more painful than the operation itself. As regards the number of operations requisite, Apostoli has found that, according to the recent or very chronic nature of the case, they vary from three to thirty, the latter figure being only very exceptionally reached.

If the patient belongs to the better classes, and is able to rest for a sufficient length of time after each seance, she may be subjected to treatment two or three times a week, but when she belongs to the working class, once or twice, should be the limit.

In concluding these remarks, I trust you will pardon me for again calling attention to the great importance of practicing strict antisepsis in all operations upon the cervix, or for that matter, upon the uterus or vagina; Apostoli lays great stress upon the observance of this rule in conducting treatment by the galvanic current after his method.

It is well to remember, that a current of electricity, when passed through a gravid womb, will certainly destroy the fœtus and put an end to the pregnancy; hence we should be certain that this condition is not present before instituting this treatment.

A safe rule is, to begin this treatment soon after menstruation has ceased, and forbid the conjugal relation during the time such treatment is practiced.

